# Manipulating Voice Attributes by Adversarial Learning of Structured Disentangled Representations

**DOI:** 10.3390/e25020375

**Published:** 2023-02-18

**Authors:** Laurent Benaroya, Nicolas Obin, Axel Roebel

**Affiliations:** Analysis/Synthesis Team—STMS, IRCAM, Sorbonne University, CNRS, French Ministry of Culture, 75004 Paris, France

**Keywords:** voice conversion, attribute manipulation, representation learning, information disentanglement, adversarial learning, cross-entropy

## Abstract

Voice conversion (VC) consists of digitally altering the voice of an individual to manipulate part of its content, primarily its identity, while maintaining the rest unchanged. Research in neural VC has accomplished considerable breakthroughs with the capacity to falsify a voice identity using a small amount of data with a highly realistic rendering. This paper goes beyond voice identity manipulation and presents an original neural architecture that allows the manipulation of voice attributes (e.g., gender and age). The proposed architecture is inspired by the fader network, transferring the same ideas to voice manipulation. The information conveyed by the speech signal is disentangled into interpretative voice attributes by means of minimizing adversarial loss to make the encoded information mutually independent while preserving the capacity to generate a speech signal from the disentangled codes. During inference for voice conversion, the disentangled voice attributes can be manipulated and the speech signal can be generated accordingly. For experimental evaluation, the proposed method is applied to the task of voice gender conversion using the freely available VCTK dataset. Quantitative measurements of mutual information between the variables of speaker identity and speaker gender show that the proposed architecture can learn gender-independent representation of speakers. Additional measurements of speaker recognition indicate that speaker identity can be recognized accurately from the gender-independent representation. Finally, a subjective experiment conducted on the task of voice gender manipulation shows that the proposed architecture can convert voice gender with very high efficiency and good naturalness.

## 1. Introduction

### 1.1. Context

Voice conversion (VC) consists of digitally altering the voice of an individual, e.g., its identity, accent, or emotion, while maintaining its linguistic content unchanged. Primarily applied to identity conversion [1,2], VC has considerably increased in both popularity and in quality thanks to the advances accomplished with neural VC; see the three editions of the VC challenge [3,4,5] for a short review of the latest challenges and contributions. Similar to face manipulation, voice conversion has a wide range of potential applications, such as voice cloning and deep faking [6] in the fields of entertainment and fraud, anonymization of voice identity [7,8] in the fields of security and data privacy, and digital voice prosthesis of impaired speech [9] in the field of digital healthcare. In its original formulation, the VC task consisted of learning the one-to-one statistical acoustic mapping between a pair of source and target speakers from a common set of temporarily pre-aligned sets of utterances [2]. During training, the joint acoustic distribution between the source and target speakers was modelled from a set of parallel utterances, usually by means of a Gaussian Mixture Model (GMM). During conversion, a linear regression was processed on this joint distribution in order to determine the voice characteristics of the target speaker conditionally to those of the source speaker. The use of the same sentences shared among speakers and the pre-alignment between them has greatly facilitated learning, as the mapping can be learned directly from this set of perfectly paired data. On the other hand, this constraint implies that training data have to be recorded explicitly for each speaker pair, which in turn increases the complexity of practical applications of the methods. From this original formulation, many advances have been proposed through years, including one-to-many, many-to-one, and many-to-many VC, in which a set of multiple speakers is used as prior knowledge to pre-train conversion functions which are then further adapted to an unseen utterance or speaker during conversion [10].

Neural VC, i.e., VC based on neural networks, was first introduced in [11], following the one-to-one and parallel VC paradigm and simply replacing GMM by NN in order to estimate the conversion function. Leveraging the successive advances that have been established in the theory and application of neural networks to natural language processing, computer vision, image processing, and speech processing [12,13,14,15,16], Neural VC has become a standard that has achieved highly realistic rendering of voice identity conversion on a small amount of data from a target voice.

### 1.2. Related Works

Through the multiple and various contributions in neural VC that have been presented over the recent years, an important progressive change in the VC paradigm can be distinguished from the initially agnostic learning of one-to-one VC using parallel datasets to today’s structured and informed learning of many-to-many VC from non-parallel datasets. Following the historical paradigm of one-to-one parallel VC, cycle-GAN and S2S with attention mechanism, VC models have been proposed to learn the acoustic mapping from pairs of sentences from source and target speakers. Inspired by [16], cycle-GAN VC [17,18,19] attempts to learn the identity conversion function in both directions through a cycle. Beyond the usual GAN losses, the cycle-consistency loss is assumed to stabilize the learning by encouraging the preservation of the linguistic content (seen as a ‘‘background”) during conversion. In S2S VC [20,21], the conversion is formulated in the form of a recurrent encoder and decoder, at the interface of which an attention mechanism [14] is used to learn the alignment between the recurrent encoding of the source and target speakers sequences, thereby optimizing the sequential learning of the conversion. However, the one-to-one VC framework using parallel datasets remains highly limited; the size of the parallel dataset is too small to efficiently learn a conversion, and there is no solution to exploit knowledge from large and non-parallel dataset to overcome this limitation.

To overcome the shortcomings of this paradigm, research efforts have gradually moved towards many-to-many and non-parallel datasets, allowing the scalability of neural VC with large and multiple speakers datasets, with the assumption that the increase of data can induce a substantial increase in the quality and naturalness of the VC. Among the first attempts, starGAN VC [22,23] was proposed to extend the paradigm of cycle-GAN to many-to-many and non-parallel VC by proposing a conditional encoder–decoder architecture. As opposed to cycleGAN VC, starGAN VC is composed of a single encoder–decoder in which the decoder is conditioned on the speaker identity to be reconstructed. In addition to the usual cycle-consistency and discriminator losses of a cycle-GAN, a classifier loss is added to determine the speaker identity from the converted speech. Further research attempted to break the need to learn any conversion function, either one-to-one or many-to-many, by formulating the VC problem as a conditional auto-encoder [24,25,26]. Similar to starGAN, this architecture is an auto-encoder in which the encoder part encodes the source speaker from the input source speaker’s utterance and the decoder part reconstructs the target speaker’s utterance from the source encoding and a speaker embedding. The fundamental difference is in the fact that during training the source and the target speakers are simply the same. During conversion, it is only necessary to manipulate the speaker attribute in the decoder to convert the input speech to the desired target identity. This breakthrough, known as few-shot [25] or zero-shot VC [26], has opened the way to high-quality VC from a very small number of examples of the target speaker.

In another line of research, VC based on comprehensively structured speech representations has been investigated. According to the fundamental model of speech communication, speech conveys verbal and non-verbal information: linguistic (the primary meaning, i.e., the text or content), para-linguistic (secondary information that helps to understand the intended meaning, e.g., the modality of a question or emotional state of the speaker), and extra-linguistic (which provides only information about the speaker, e.g., their identity or socio-geographical origin). VC architectures have started to integrate explicitly linguistic content and speaker identity [27] information, e.g., by the explicit use of textual information (Phonetic Posterior-Grams, PPG [28,29]) and with speaker representation, usually referred to as speaker embedding [30]. In order to efficiently learn a structured representation from raw data, it is necessary to disentangle the information encoded in the signal [31]. This problem can be written in the form of a neural network and tackled by adopting an information bottleneck [32] or adversarial [12,33] strategy, both of which are grounded in information theory [34]. In [35], three bottlenecks were used to separately encode the speech parameters of pitch, timbre, and rhythm, while in [36,37] the authors employed adversarial learning of disentangled representations to learn a set of representations that encodes specifically linguistic information and speaker identity, ideally independent from one another. While recent VC systems can achieve realistic voice identity conversion with limited data of the target speaker, in contrast to the wealth of research into the manipulation of face attributes [33,38,39] there do not exist many studies that investigate the conversion of other voice attributes, such as, for example, age and gender.

### 1.3. Contributions of This Paper

This paper proposes a structured neural VC architecture for manipulating voice attributes by means of disentangling the attributes in the latent representation. The main contributions of the paper can be listed as follows:

- An extension of the VC architecture presented in [40] that allows for the encoding and manipulation of the voice by means of multiple attributes (content, identity, age, or gender);
- An implementation of a network for voice attribute disentanglement based on a fader network [33], an adversarial neural network originally established for face manipulation. In the proposed VC architecture, the speaker identity code is further decomposed adversarially into two parts, namely, a speaker identity code that is independent of the desired attribute and an attribute code;
- The application of the proposed neural architecture to voice gender manipulation. While this study only focuses on voice gender manipulation, we foresee extending it in the future to manipulate other identity-related attributes such as age, accent, or speaking style.

The remainder of this paper is organized as follows: Section 2 presents the core VC architecture and the proposed neural disentanglement strategy, while Section 4 presents a complete experimental evaluation of the proposed VC architecture with application to voice gender manipulation, including objective and subjective experiments.

## 2. Neural VC: Manipulating Identity and Beyond

### 2.1. Neural VC with Content and Identity Disentanglement

**Figure 1:**
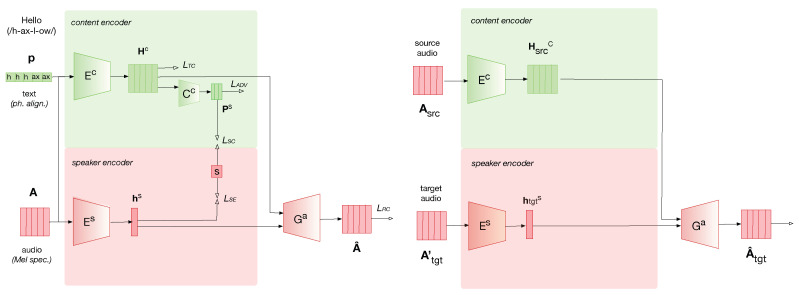
Architecture of the neural VC system with adversarial learning of disentangled linguistic and speaker representation. Top: training phase. Bottom: conversion phase. See Section 2.1 for detailed description.

The neural VC architecture used in this paper is based on the architecture presented in [40], which was inspired by [36]. The main idea of this VC architecture is that disentangled linguistic and speaker representation are encoded adversarially through dedicated encoders, as illustrated in Figure 1. The inputs of the VC architecture are the speech signal matrix A, represented by the mel-spectrogram computed on *T* time frames, and the sequence of *T* phonemes p corresponding to the phonetic transcription of the input text aligned to the corresponding speech signal. Dual encoders, $E^{c}$ and $E^{s}$, are employed to encode linguistic content and speaker information.

#### 2.1.1. Speaker Encoder

The speaker encoder $E^{s}$ converts the speech signal A into a time-independent vector $h^{s}$, as it is assumed that the identity of a speaker does not vary within an utterance:

(1) $$h^{s}=E^{s}\left(A\right)$$

The speaker classification loss $L_{SE}$ is defined as the cross-entropy between the speaker identity predicted from $h^{s}$ by a classifier $C_{s}^{s}$, and the true speaker identity s encoded in the form of a one-hot vector

(2) $$L_{SE}\left(C_{s}^{s}|E^{s}\right)=E_{A}\;CE\left(C_{s}^{s}\left(h^{s}\right),s\right)$$

where CE(.,.) denotes the cross-entropy between two random variables. Please note that the cross-entropy can be interpreted directly in terms of the Kullback–Leibler divergence between the distributions of the two considered variables, i.e., the extra quantity of information needed to code the true distribution when using the priors of the estimated distribution. In particular, the cross-entropy with softmax activation can be interpreted directly in terms of mutual information between true labels and predicted labels in the case of a classification task [41]. This indicates a strong interlacing between neural networks and information processing in light of information theory.

#### 2.1.2. Content Encoder

The content encoder $E^{c}$ converts either the phoneme sequence p or the speech signal A into a shared linguistic embedding $H^{c}$ through a contrastive loss (see [36] for details):

(3) $$H^{c}=E^{c}\left(A\right)$$

Learning a shared encoding among both audio and text modalities can be related to cross-modal domain adaptation, in which one wants to learn a code that is independent of the input modality or distribution. As a result, the content encoder is trained to transcribe the phonetic content from the speech signal. In this paper, the linguistic embedding has the same length *T* as the aligned phoneme sequence (as well as the mel-spectrogram), meaning that the time information is fully preserved during encoding.

The content recognition loss $L_{TC}$ is defined as the cross-entropy between the phoneme predicted from $h_{n}^{c}$ by the classifier $C^{c}$ and the corresponding true phoneme label $p_{n}$ for the *n*th time frame:

(4) $$L_{TC}\left(C^{c}|E^{c}\right)=E_{p}\;CE\left(C^{c}\left(h_{n}^{c}\right),p_{n}\right).$$

#### 2.1.3. Disentangling Identity and Content Information

In order to disentangle content and identity information, an adversarial strategy is added to remove identity information from the linguistic embedding $H^{c}$. The speaker classifier loss is defined as the cross-entropy between the speaker identity predicted from $h_{n}^{c}$ by the classifier $C_{s}^{c}$ and the true speaker identity s. An adversarial loss $L_{ADV}\left(E^{c}\right)$ is additionally defined with the opposite objective of learning linguistic representation $H^{c}$, from which the speaker identity can not be recognized by the speaker classifier:

(5) $$L_{ADV}\left(E^{c}|C_{s}^{c}\right)=E_{A}\;||u-C_{s}^{c}\left(h_{n}^{c}\right){\left|\right|}_{2}^{2}$$

where *u* denotes a uniform distribution in which all speakers have the same probability 1/S, with *S* being the total number of speakers in the dataset.

#### 2.1.4. Decoder

A decoder $G^{a}$ conditioned on the disentangled content embedding $H^{c}$ and speaker embedding $h^{s}$ is employed to reconstruct an approximation $\hat{A}$ of the original speech signal A:

(6) $$\hat{A}=G^{a}\left(h^{s}=E^{s}\left(A\right),H^{c}=E^{c}\left(A\right)\right)$$

A reconstruction loss $L_{RC}$ is defined between the mel spectrogram of the reconstructed speech signal $\hat{A}$ and the mel spectrogram of the original speech signal A.

(7) $$L_{RC}\left(E^{s},E^{c},G^{a}\right)=E_{A}\;||G^{a}\left(E^{s}\left(A\right),E^{c}\left(A\right)\right)-A{\left|\right|}_{1}$$

During training, the VC neural network is pre-trained on a dataset containing multiple speakers. As the VC architecture mainly relies on an auto-encoder, there is no attribute manipulation or conversion during training. This limitation has been further addressed in [40] During conversion, the content encoder $E^{c}$ computes the content embedding $H_{src}^{c}$, corresponding to one utterance $A_{src}$ of the source speaker, solely from the audio modality, as follows: $H_{src}^{c}=E^{c}\left(A_{src}\right)$. Meanwhile, the speaker encoder $E^{s}$ computes the speaker embedding $h_{tgt}^{s}$ corresponding to one utterance $A_{tgt}^{\prime }$ of the target speaker, as follows: $h_{tgt}^{s}=E^{s}\left(A_{tgt}^{\prime }\right)$. Then, the decoder $G_{a}$ is conditioned on the linguistic embedding $H_{src}^{c}$ and the speaker embedding $h_{tgt}^{s}$ to generate the utterance ${\hat{A}}_{tgt}$ with the identity of the target speaker, ${\hat{A}}_{tgt}=G_{a}\left(h_{tgt}^{s}=E^{s}\left(A_{tgt}^{\prime }\right),H_{src}^{c}=E^{c}\left(A_{src}\right)\right)$. In this way, an utterance with the linguistic content of the source utterance is pronounced with the identity of the target speaker.

### 2.2. Disentanglement of Voice Attributes with Fader Network

**Figure 2:**
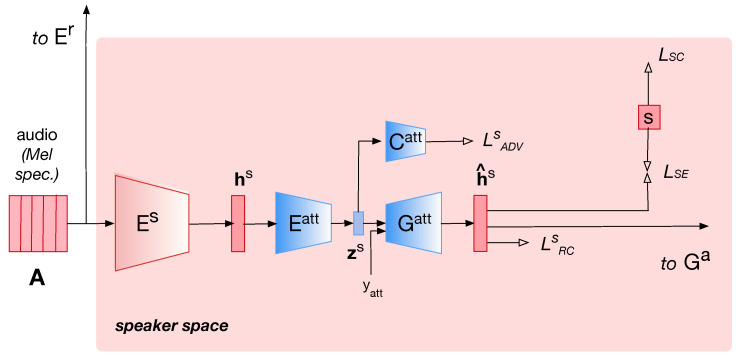
Architecture of the proposed speaker disentanglement. The speaker code $h^{s}$ is disentangled into an attribute code att and a speaker code $z^{s}$ that are independent on attribute att. For simplicity, only the speaker space of the architecture is presented.

In the previous section, we presented the disentanglement of speech content and speaker identity which is processed adversarially between parallel encoding. In the present section, we introduce further disentanglement of voice attributes by proposing cascade disentanglement using a fader network [33], as illustrated in Figure 2.

The speaker embedding $h^{s}$ resulting from the speaker encoder $E^{s}$ in the speaker space serves as the input of the proposed fader network. This fader network is an autoencoder in which the speaker embedding is encoded by $E^{att}$ to a low-dimensional latent code $z^{s}$.

(8) $$z^{s}=E^{att}\left(h^{s}\right)$$

Conversely, the decoder $G^{att}$ tries to reconstruct the speaker embedding ${\hat{h}}^{s}$ from the latent code $z^{s}$ and the conditioning attribute variable $y_{att}$.

(9) $${\hat{h}}^{s}=G^{att}\left(z^{s},y_{att}\right)$$

The objective of the fader network is to be able to reconstruct the input variable ${\hat{h}}^{s}$ from the latent code $z^{s}$ and the conditioning variable $y_{att}$. To ensure that the conditioning variable is effective, the goal is to make the latent code $z^{s}$ independent on the conditioning variable $y_{att}$. To do this, we employ an adversarial scheme.

First, the reconstruction loss of the auto-encoder $L_{RC}^{S}$ is defined as the mean absolute error between the speaker embedding $h^{s}$ and the reconstructed speaker embedding ${\hat{h}}^{s}$:

(10) $$\begin{array}{c} L_{RC}^{S}\left(E^{att},G^{att}\right)=E_{h^{S}}\;\;{∥h^{S}-G^{att}\left(E^{att}\left(h^{S}\right),y_{att}\right)∥}_{1} \end{array}$$

The objective of this first loss is that the encoder $E^{att}$ encodes the information $z^{s}$ in such a way that the decoder $G^{att}$ is able to reconstruct the original input from the latent code $z^{s}$ and the conditioning attribute $y_{att}$. 

Second, a discriminator loss $L_{D}^{S}$ is defined as the cross-entropy between the attribute predicted by the classifier $C^{att}$ and the true attribute $y_{att}$, represented in the form of a one-hot vector:

(11) $$\begin{array}{c} L_{D}^{S}\left(C^{att}|E^{att}\right)=E_{h^{S}}\;\;CE\left(y_{att},C^{att}\left(E^{att}\left(h^{S}\right)\right)\right) \end{array}$$

The objective of this second loss is that the classifier $C^{att}$ is able to predict the correct attribute $y_{att}$ from the latent code $z^{s}$. 

Third, an adversarial loss $L_{ADV}^{S}$ is defined as the cross-entropy between the attribute predicted by the classifier $C^{att}$ and the wrong attribute $1-y_{att}$, as follows:

(12) $$\begin{array}{c} L_{ADV}^{S}\left(E^{att}|C^{att}\right)=E_{h^{S}}\;\;CE\left(1-y_{att},C^{att}\left(E^{att}\left(h^{S}\right)\right)\right). \end{array}$$

The objective of this loss is that the classifier $C^{att}$ cannot predict the the correct attribute $y_{att}$ from the latent code $z^{s}$. This is defined in order to make the latent code $z^{s}$ independent on the $y_{att}$ variable. 

Finally, the total adversarial loss of the fader network can be written as

(13) $$\begin{array}{c} L_{RC}^{S}\left(E^{att},G^{att}|C^{att}\right)=L_{RC}^{S}\left(E^{att},G^{att}\right)+\lambda L_{ADV}^{S}\left(E^{att}|C^{att}\right). \end{array}$$

In this paper, $y_{att}$ encodes the gender of the speaker as $y_{att}=0.0$ for female and $y_{att}=1.0$ for male. Additionally, the attribute discriminator $C^{att}$ tries to predict the attribute $y_{att}$ from the latent code $z^{s}$. A discriminator that is pre-trained on the speaker embedding $h^{s}$ is employed to substitute the binary attribute $y_{att}\in \left\{0,1\right\}$ by the smooth posterior probability of the discriminator ${\tilde{y}}_{att}\in \left[0,1\right]$. Finally this fader is directly plugged into the speaker space of the VC system after the speaker encoder $E^{s}$. It is then possible to retrain the decoder $G^{a}$ of the global VC system, which we describe in one of the configurations in the experimental section. The proposed architecture is acoustically agnostic; the network learns voice attribute codes directly from the mel-spectrogram representation of the speech signal without any assumptions about the acoustic characteristics being used to encode one particular voice attribute. For instance, $h^{s}$ encodes all the time-fixed information related to the speaker’s identity, which we assume includes its gender. Then, $y_{s}$ is a binary code exclusively representing the gender of the speaker and $z^{s}$ encodes the speaker’s identity independently of gender. Finally, the decoder $G^{att}$ which reconstructs the speech signal from the latent speech representation learns a mapping between the disentangled codes and their actual acoustic characteristics by mean of a mel-spectrogram representation.

## 3. Implementation Details

### 3.1. Neural VC Architecture

The model configuration parameters are the same as those described in [36], with the exception of the recognition encoder $E^{r}$ and the decoder $G^{a}$ (referred to as $D^{a}$ in [36]), which are modified for the time-synchronized VC system. Table 1 presents the details of these modification, together with the components of the fader network used for identity and gender disentanglement, namely, the encoder $E^{att}$, the classifier $C^{att}$, and the decoder $G^{att}$. The simplifications realized with respect to the recognition encoder $E^{r}$ and the decoder $G^{a}$ enable time-synchronous conversions and consequent savings in computation time equating to approximately 33% of the total computation time for training on our server with a single GPU GForce GFX 1080Ti.

### 3.2. Pre- and Post-Processing

Following [36], our system operates on a mel-spectrogram representation of the speech signal. For the signal analysis we follow the parameterization proposed in [42], that, is the input signal is downsampled to 16 kHz, then converted into an STFT using a Hanning window of 50 ms with hop size of 12.5 ms and an FFT size of 2048. We then use 80 mel bins covering the frequency band from 0 to 8 Khz and convert the result into the log amplitude domain. A standardization of the log-mel-spectrogram is applied at the input of the VC system, i.e., on each mel bin, removing the mean and diving by the standard deviation, which are pre-computed on the entire training dataset. A multi-speaker approach is required for rendering audio from a generated mel spectrogram, as the generated mel spectrograms are not linked to any existing speaker identity. We initially used a Griffin and Lim [43] algorithm for phase reconstruction; however, this did not provide sufficient quality for perceptual evaluations. We then resorted to a multi-speaker waveglow-type decoder, loosely following [44]. This decoder was trained over 900,000 iterations using all samples of the VCTK database with a batch size of 50 and segment length of 375 ms and using the Adam optimizer with a learning rate of $10^{-4}$. While the quality of this decoder is far from perfect, it provided consistently better results than the quality obtained with the Griffin and Lim algorithm, and was used for the perceptual tests. The decoder has a slight tendency to produce an overly rough voice quality, indicating instability on F0. The decoder is subject to further research, and will be published elsewhere.

### 3.3. Computation Infrastructure and Runtime Costs

All training runs were performed on a single GPU (GForce GFX 1080Ti). The inference and the mel inversion were run on the CPU (Xeon(R) CPU E5-2630 v4 @ 2.20 GHz), while a number of the figures have been generated using the GPU. The duration of the VC model training is 20 min per epoch with 80 epochs (roughly 27 h), and the training of the gender autoencoder model lasts 1 min and 30 s per epoch with 400 epochs (total of 10 h). The inference of one sentence of 1.5 s takes around 2 s for computing the mel-spectrogram plus two seconds for mel inversion when using our CPU.

With respect to the training parameters, the VC system makes use of the Adam optimizer with a learning rate equal to $10^{-3}$ and a batch size of 32, while training of the gender autoencoder is carried out with the SGD optimizer using a learning rate equal to $10^{-4}$, with the momentums set to 0.9 and a batch size of 64. In addition, the pre-trained gender discriminator makes use of the SGD optimizer, again with a learning rate equal to $10^{-4}$ and momentum of 0.9; three epochs are used, with each epoch lasting 1 min and 30 s, and the batch size is equal to 64.

## 4. Experiments

### 4.1. Dataset

The English multi-speaker VCTK corpus [45] is used for VC and gender model training as well as for gender conversion. The VCTK dataset contains speech data uttered by 110 speakers and the corresponding text transcripts. Each speaker reads about 400 sentences selected from English newspaper, which represents a total of about 44 hours of speech. All speakers were included in the training and validation sets. For each speaker, we split the database into a training set with 90% of the sentences and a validation set with 10% of them. The total duration of the database was around 27 h after removing silences at the beginning and end of each sentence.

### 4.2. Preliminary Illustration

**Figure 3:**
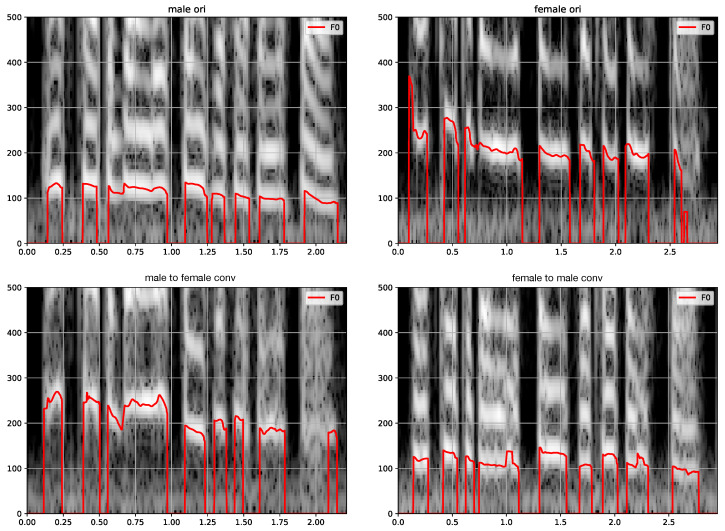
Visualization of the spectrograms and F0 curves of the sentence “Ask her to bring these things with her from the store.” **Top**: Two spectrograms of original speech signals of a male speaker (**left**) and a female speaker (**right**). **Bottom**: Spectrograms of the two signals after gender conversion using the proposed model. The solid red line is the F0. The y-axis shows the frequency in Hertz, while the x-axis shows the time in seconds.

Figure 3 shows four spectrograms superimposed with related pitch contours (F0, in red solid lines). The sentence “*Ask her to bring these things with her from the store*” is uttered by a male speaker (left) and by a female speaker (right). The top figures show the original signals and the bottom figures correspond to the conversion conditioned on the opposite gender. The gender conversion algorithm clearly transposes the average F0 in line with what we would have done to convert between male and female speakers using traditional vocoders (±1 octave) [46]. However, in contrast to what we would have done when using traditional vocoders, here the transposition is dynamic, changing the intonation contours as well. Additionally, the algorithm creates vocal fry at the final words of the utterance when converting from male to female, while it does the opposite when converting from a female to a male voice. We conjecture that this presence or absence of vocal fry reflects a general tendency of the male and female voices in the database.

### 4.3. Objective Evaluations

To assess whether the proposed framework is successful in disentangling speaker identity and gender representation, a set of objective evaluations were conducted: a gender classification task (including a short ablation study on the fader structure), a speaker classification task, the mutual information between the embeddings and the true gender, and a 2D visualization of the embeddings.

#### 4.3.1. Experiment 1: Gender Recognition

Table 2 reports the gender classification accuracy computed with the pre-trained gender discriminator at the original speaker embedding $h^{s}$ (*original*) or the reconstructed speaker embedding ${\hat{h}}^{s}$ of the gender autoencoder with different values of gender conditioning *w*: with the estimated gender $\hat{w}$ from the original speech signal (*est. gender*), by swapping to the opposite gender $1-\hat{w}$ (*inv. gender*), or by neutralizing the gender 1/2 (*de-gender*). With the adversarial setting, the original speaker embedding and the reconstructed speaker embedding with the estimated gender have very high accuracies. When swapping the gender by conditioning the reconstructed embedding on the opposite of the estimated gender the accuracy becomes zero, which is expected because the gender is inverted. With reconstruction conditioned on 1/2, the accuracy is around 50%, which corresponds to a random decision in a binary classification problem. In the ablation study conducted by removing the adversarial component from the fader network, the accuracies are very high in all conditions, which means that the gender conditioning is ineffective. Therefore, the adversarial loss is necessary for disentangling the speaker’s gender from the speaker’s identity. This shows that the adversarial loss is both required and highly efficient for disentangling and manipulating speaker gender with respect to speaker identity.

#### 4.3.2. Experiment 2: Speaker Recognition

**Figure 4:**
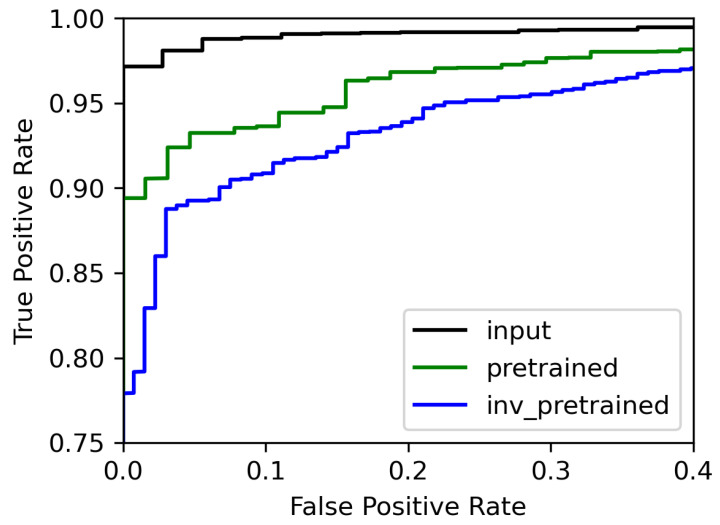
Receiver Operating Characteristic curves of speaker classification using the speaker encoder classifier as computed on the original speaker embedding $h^{s}$ and the reconstructed speaker embedding conditioned on the gender *w*. In black, *w* is the actual binary gender; green is the gender as classified by the pre-trained gender classifier $w=\tilde{w}$; finally, blue is the inverse gender as classified by the pre-trained gender $w=1-\tilde{w}$.

A Receiver Operating Characteristic curve, or ROC curve, is a graphical plot that illustrates the diagnostic ability of a binary classifier as its discrimination threshold is varied. The ROC curve is created by plotting the true positive rate (TPR) against the false positive rate (FPR) at various threshold settings. The Equal Error Rate (EER) is the error rate of a binary classifier when the operating threshold for the accept/reject decision is adjusted such that the probability of false acceptance and that of false rejection become equal. On the ROC curve, it corresponds to the intersection with the anti-diagonal line. Figure 4 presents the Receiver Operation Characteristic (ROC) curves corresponding to the speaker classification from the original speaker embedding and the reconstructed speaker embedding conditioned on gender, while Table 3 summarizes the equal error rates (EERs) obtained from the original speaker embedding and the reconstructed speaker embedding conditioned on gender. The EER is very low (2.8%) for the original speaker embedding, which indicates that the speaker classifier is very efficient at determining speaker identity from the speaker embedding. Manipulation of the gender conditioning *w* degrades the EER in all cases; however, these rates remain relatively low, at around 6.8% for the gender estimated from the pre-trained classifier and w=1/2 and around 9.2% when the gender is swapped. This means that most of the speaker identity is preserved after gender manipulation. However, the speaker identity cannot be totally preserved, as identity and gender are certainly not linearly separable variables.

#### 4.3.3. Experiment 3: Mutual Information and Visualization of Embeddings

**Figure 5:**
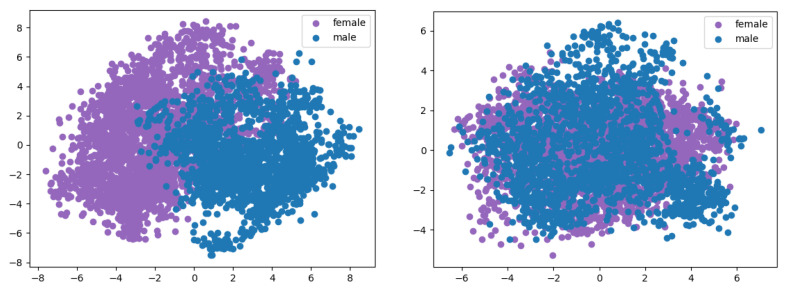
**Left**: PCA visualization of the speaker encoder embeddings $h^{s}$ on the evaluation set; the selected components are 1 and 2. **Right**: PCA visualization of the latent code $z^{s}$ on the evaluation set; the selected components are 3 and 7.

Table 4 presents the approximated calculation of the mutual information between the true gender and the original speaker embedding and the conditionally reconstructed speaker embeddings. This score is computed using an estimator of the mutual information between discrete and continuous variables, as described in [47]. The dimension of the continuous data is reduced from 128 to 8 using PCA and the mutual information is obtained by selecting the pair of coordinates that maximize the latter. The PCA coordinates used to plot the 2D visualizations in Figure 5 are selected in the same way. From Table 4, the mutual information corresponding to the latent code $z^{s}$ and the de-gender w=1/2 are much lower that the others. This indicates that the latent code $z^{s}$ contains very little information about the gender and becomes mostly gender-independent, as illustrated in Figure 5, as well as that the conditioning w=1/2 successfully generates a speaker embedding that is mostly genderless. This highlights the fact that the proposed method for achieving disentanglement between speaker identity and gender is highly effective.

### 4.4. Subjective Evaluation

To assess whether the proposed architecture is efficient at converting the gender of the voice, a subjective evaluation was conducted.

#### 4.4.1. Baseline Algorithm

To the best of our knowledge, there are no neural gender conversion algorithms available in the literature; therefore, we used a traditional signal processing approach as our baseline for perceptual tests. Classic voice transformation algorithms perform gender manipulation by means of modifying the average of the fundamental frequency (F0) and the positions of the vocal tract resonances (known as formants). Due to physiological differences between the female and male voice organs, notably the size of the vocal folds and vocal tract, these two parameters have average values which generally differ for male and female voices. These differences have been measured and documented in the literature [48,49]. Considering that these parameters are part of the physiological configurations of the vocal organs, they are part of the speaker’s identity; it has been shown in [46] that a constant and independent transposition of the F0 and the formants can be used to successfully modify the perceived gender and age of a voice. Following these findings, we use the following parameters for gender conversion: F0 is shifted by ± one octave (±1200 cents) and the spectral envelope is shifted by ±3 semi-tones (i.e., ±300 cents), where the sign of the shift depends on the gender of the original sound. For male to female, a positive sign is used, while a negative sign is used for female to male conversion. A shape-invariant phase vocoder [50] is used for signal manipulation by using the true envelope estimator for the representation of the formant structure [51]. These types of algorithms have been used successfully in the past for gender transformation for professional uses. However, the default setup does not work equally well for all voices, and manual fine tuning is generally employed to optimize the coherence of the transformed voice signal. As the proposed algorithm is fully automatic, we did not apply manual tuning for the signals used in the subjective tests.

#### 4.4.2. Experimental Protocol

The task consisted of listening to one speech sample (converted or not) and judging the following:

(1) whether the voice is typically perceived as: *feminine*, *rather feminine*, *uncertain*, *rather masculine*, or *masculine*;
(2) the sound quality on a standard Mean Opinion Score (MOS) 5-degree scale from 1 (*bad*) to 5 (*perfect*), which is commonly used for experimental evaluation of Text-To-Speech and Voice Conversion systems.

**Figure 6:**
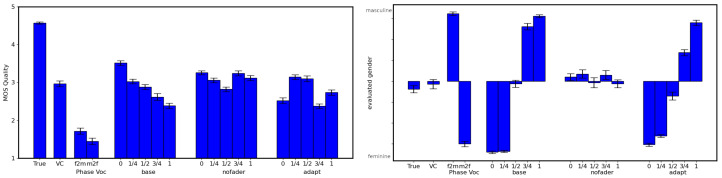
**Left**: MOS scores obtained for the six configurations (mean and 95% confidence interval). **Right**: perceived voice gender for the six configurations (mean and 95% confidence interval).

Each participant had to judge twenty speech samples which were randomly selected from among all of the speech samples produced for the subjective experiments. Four speakers were used for the experiment, two males (p232 and p274) and two females (p253 and p300), with five randomly chosen sentences per speaker in the validation set. Six configurations were compared (the term in parenthesis is used as an identifier in Figure 6):

(1) the original audio signal (*True*) and converted audio signal with:
(2) the original VC system (*VC*);
(3) a phase vocoder (*phase voc.*; see supplementary for details) with two cases: female-to-male conversion (f2m) and male-to-female conversion (m2f);
(4) the VC system with the proposed gender autoencoder (*base*) with five conditioning values of the parameter $\tilde{w}\in \left\{0,1/4,1/2,3/4,1\right\}$;
(5) the VC system with the gender autoencoder but trained without the fader loss (*nofader*), with the five values of the $\tilde{w}$ parameter; and
(6) the VC system with the gender autoencoder with the VC decoder re-trained (*adapt*) with the five values of the $\tilde{w}$ parameter.

#### 4.4.3. Results and Discussion

Figure 6 presents the MOS scores and perceived gender for the compared system configurations (mean and 95% confidence interval). Regarding the perceived quality, the original speech samples have the highest score (4.6), the original VC system samples have similar scores as the ones reported in [36] (2.90), and the samples converted with the phase vocoder have fairly low scores (1.6), which is due to the use of the default settings and indicates the limitation of voice conversion based on signal processing only. The three versions of our proposed VC system have similar scores that are comparable to those of the original system (between 3.0 and 4.0): 2.9 for the *base* VC system, 3.11 for the *nofader* VC system, and 2.97 for the *adapt* VC system. This shows that the addition of the gender auto-encoder does not degrade the conversion quality. While MOS scores do not constitute a direct measurement of speech intelligibility, the perceived quality of the speech signal clearly is an indicator of speech intelligibility. The scores that we obtained show that the proposed VC has a high rendering sound quality. This quality tends to be degraded in the case of the *base* VC system from female to male; however, this trend tends to disappear for the *adapt* VC system in which the VC decoder is re-trained together with the gender auto-encoder. Regarding the perceived gender, the true gender is easily recognized for the original speech samples, the converted speech with the original VC system, and the converted speech with the phase vocoder. As mentioned previously, the VC system with a gender autoencoder without fader loss is totally inefficient at converting the gender. For the proposed VC system with gender auto-encoder, the gender conditioning is efficient at manipulating the perceived gender during conversion, as a clear variation of the perceived gender can be observed with respect to the conditioned gender. In the *base* VC system, however, there is a discontinuity around the value w=1/2, which means that the conversion jumps from female to male and fails to generate genderless voices. This appears to be much more linear in the *adapt* VC system, which again indicates that the re-training of the VC decoder can improve conversion around the genderless value (w=1/2).

## 5. Conclusions

This paper presents a structured neural VC architecture that allows the manipulation of voice attributes (e.g., gender and age) based on adversarial learning of a hierarchically structured speech and speaker encoding. The proposed VC architecture employs multiple auto-encoders to encode speech as a set of idealistically independent linguistic and extra-linguistic representations, which are learned adversarially and can be manipulated during VC. Moreover, the proposed architecture is time-synchronized, meaning that the original voice timing is preserved during conversion; this enables its use in lip-syncing applications. A set of objective and subjective evaluations conducted on the VCTK dataset shows the efficiency of the proposed framework on the task of voice gender manipulation. Our further work will investigate the generalization of the proposed framework to other voice attributes, such as age, attitude, and emotion.

## Figures and Tables

**Table 1:** Model configuration details. FC refers to a fully-connected layer, BLSTM to a bi-directional LSTM, and Tanh to the hyperbolic tangent activation function. The right arrow → indicates successive layers in the network.

$E^{r}$	2 layers BLSTM-Dropout(0.2), 256 cells each direction → FC-512-Tanh
$E^{s}$	2 layers BLSTM-Dropout(0.2), 128 cells each direction → average pooling → FC-128-Tanh
$G^{a}$	2 layers BLSTM, 64 cells each direction → FC-80
$E^{att}$	FC-60
$G^{att}$	FC-1
$G^{att}$	FC-128-Tanh

**Table 2:** Ablation study: gender classification accuracy using the pre-trained discriminator computed on the original speaker embedding $h^{s}$ and the reconstructed speaker embedding conditioned on the gender *w*. The dimension of the speaker embedding $h^{s}$ is 128 and the dimension of the latent code $z^{s}$ to 60.

	Gender Accuracy [%]
*with adv. loss*	
Original $h^{s}$	99.2
Est. Gender ($w=\tilde{w}$)	99.0
Inv. Gender ($w=1-\tilde{w}$)	0.8
De-gender (w=1/2)	54.6
*without adv. loss*	
Original $h^{s}$	99.2
Est. Gender ($w=\tilde{w}$)	99.2
Inv. Gender ($w=1-\tilde{w}$)	98.8
De-gender (w=1/2)	99.1

**Table 3:** Equal Error Rates in percentages of speaker classification using speaker encoder classifier computed on the original speaker embedding $h^{s}$ and the reconstructed speaker embedding conditioned on the gender *w*. The dimension of the speaker embedding $h^{s}$ is 128 and the dimension of the latent code $z^{s}$ is 60.

	EER [%]
Original $h^{s}$	2.8
Est. Gender ($w=\tilde{w}$)	6.9
Inv. Gender ($w=1-\tilde{w}$)	9.2
De-gender (w=1/2)	6.8

**Table 4:** Approximation of the mutual information between the true gender and the continuous multi-dimensional embedding. The dimension of the speaker embedding $h^{s}$ is 128 and the dimension of the latent code $z^{s}$ is 60.

	Mutual Information
Original $h^{s}$	0.47
Est. Gender ($w=\tilde{w}$)	0.44
Inv. Gender ($w=1-\tilde{w}$)	0.38
De-gender (w=1/2)	0.16
Latent code $z^{s}$	0.11
